# Decline in the Conception Rate of Wild Japanese Monkeys after the Fukushima Daiichi Nuclear Power Plant Accident

**DOI:** 10.1007/s00244-024-01063-z

**Published:** 2024-04-25

**Authors:** Shin-ichi Hayama, Setsuko Nakanishi, Aki Tanaka, Takuya Kato, Chinatsu Watanabe, Nobutaka Kikuchi, Risa Danjo, Ayano Matsuda, Wakako Mori, Yuki Kawabata, Hikari Akiba, Fumiharu Konno, Yoshi Kawamoto, Toshinori Omi

**Affiliations:** 1 School of Veterinary Medicine, Nippon Veterinary and Life Science University, Musashino, Tokyo 180-8602 Japan; 2 Tohoku Wildlife Management Center, Sendai, Miyagi 989-3212 Japan; 3 School of Veterinary Nursing and Technology, Nippon Veterinary and Life Science University, Musashino, Tokyo 180-8602 Japan

## Abstract

We examined the conception rate of wild Japanese monkeys (*Macaca fuscata*) in Fukushima City that were exposed to radiation as a result of the Fukushima Daiichi Nuclear Power Plant accident in March 2011. The conception rate in the year of delivery from 2009 to 2022 was estimated by dissecting individuals that were euthanized by the government for population control as a countermeasure against crop damage. To evaluate the effects of exposure, the cumulative exposure dose for each individual was calculated using the concentration of radiocesium deposited in the soil at the capture site and the concentration of radiocesium in muscle estimated from the aggregated transfer factor. There were no significant differences in conception rates across all age classes over time. In terms of conception rates by age class, there was a significant decrease post-exposure compared with pre-exposure in the age class ≥ 8 years, but no significant differences in the age class 5–7 years. The non-ovulation rate did not significantly differ between the pre- and post-exposure periods for any age class. Body fat index, which can affect fertility, was compared between the pre- and post-exposure periods, and no significant differences were found in either age class. In contrast, the median total cumulative exposure (cumulative internal exposure + cumulative external exposure) was significantly higher in the age class ≥ 8 years compared with the age class 5–7 years. These results suggest that the total cumulative exposure dose may be one of the reasons for the lower conception rate in the post-exposure period among the age class ≥ 8 years.

Wild Japanese monkeys (*Macaca fuscata*) in the Tohoku region, which includes Fukushima City, were once threatened with extinction, and their capture has long been regulated for this reason. As a result, the population has gradually recovered and was removed from Japan’s Red List in 2007. However, as the population recovered, crop damage also increased. Therefore, in 2007, Fukushima Prefecture established a management plan based on the law and began efforts to control the Japanese monkey population with the aim of reducing damage to crops. Due to concerns that the population control measures might increase their risk of extinction once again, it became necessary to carefully monitor the age structure and conception rate of captured animals while implementing population control measures.

It was estimated that approximately 2,000 Japanese monkeys were living in Fukushima City as of 2007, and it was decided to reduce the population by about 100 to 150 monkeys per year based on the management plan of Fukushima Prefecture. Our research group, in cooperation with Fukushima City, initiated a monitoring survey in 2008 that involves dissecting all captured individuals and recording data such as sex, age, nutritional status, and conception rate, with the aim of confirming that appropriate population control is being carried out.

In March 2011, three years into the monitoring survey, an accident occurred at the Fukushima Daiichi Nuclear Power Plant (FDNPP), and Japanese monkeys in Fukushima City, which is about 70 km from the FDNPP, were contaminated by radioactive materials (Fig. 1). So far, our research group has reported phenomena such as decreased blood cell counts and delayed fetal growth in these monkeys, which are thought to be health effects caused by this radiation exposure (Ochiai et al. 2014; Hayama et al. 2017, 2023). However, we have not examined the effects of radiation exposure on their reproduction.

**Fig. 1:**
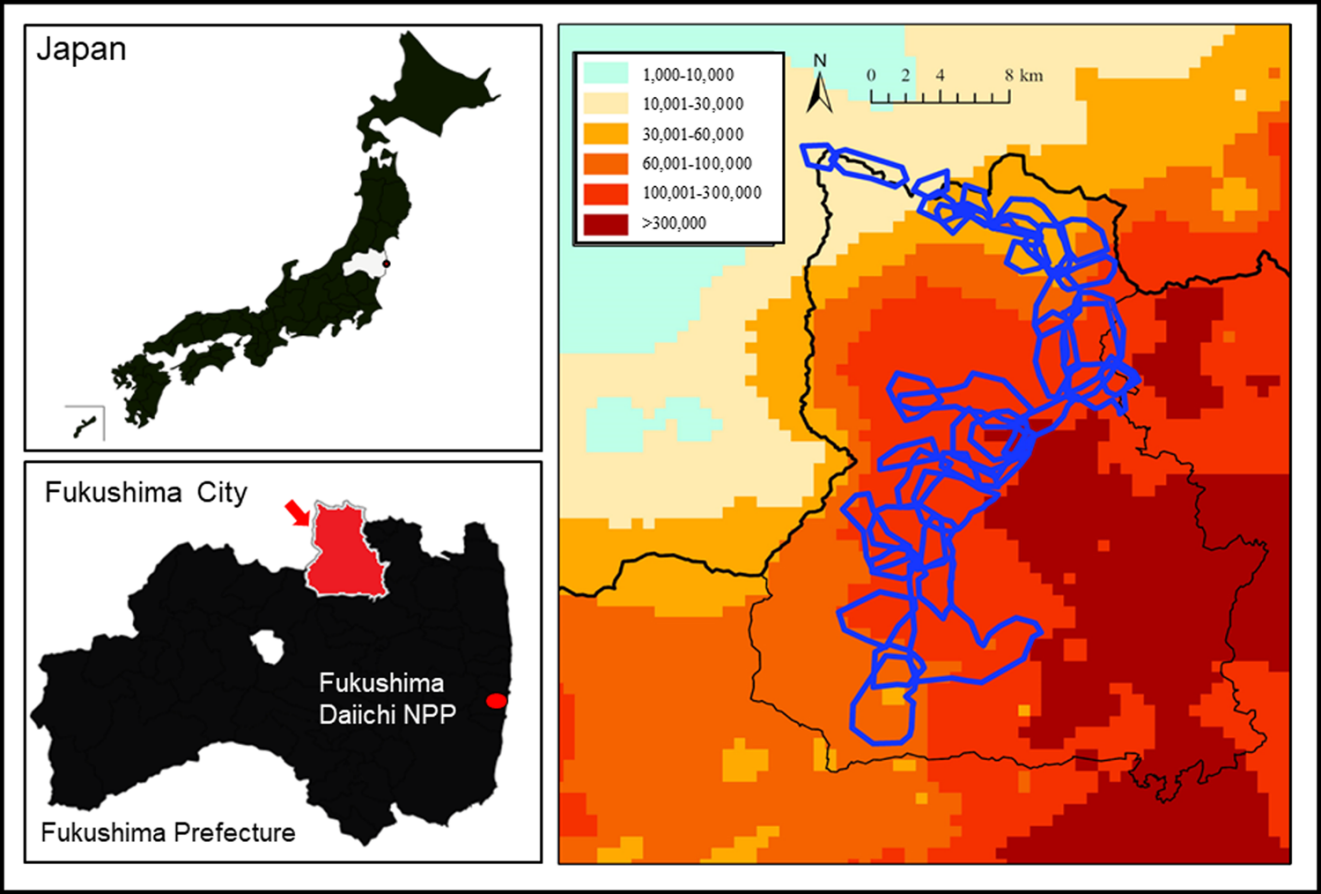
Soil contamination levels by radiocesium concentrations (Bq/m.^2^) and the distribution of monkey troops (irregular enclosed blue outlines) in Fukushima City. This map was created based on the soil contamination map created by the Ministry of Education, Culture, Sports, Science and Technology (converted to values for July 2, 2011)

Since the nuclear disasters in Chernobyl and Fukushima, many studies on human fertility and perinatal mortality have been reported, with some papers suggesting negative effects of radiation exposure (Kulakov et al. 1993; Kordysh et al. 1995; Scherb et al. 2016; Cwikel et al. 2020). However, not many studies have explored the reproductive effects of radiation exposure in domestic or wild animals (Ryabokon and Goncharova 2006; Abe et al. 2020), and few studies have suggested clear effects (Murase et al. 2015).

In the present study, to clarify the effects of exposure on reproduction in Japanese macaques in Fukushima City, we examined temporal trends in conception rates estimated from captured individuals as well as the relationship between age and exposure.

## Materials and Methods

### Animals and Ethics

This study was approved by the Institutional Animal Care and Use Committee of Nippon Veterinary and Life Science University (No. 2022S-1). All experiments were conducted in accordance with relevant guidelines and regulations. Carcasses of Japanese monkeys were provided by Fukushima City. Monkeys were captured with the permission of the governor of Fukushima Prefecture in order to prevent crop damage, in accordance with the Fukushima Japanese Monkey Management Plan, which was established based on the Wildlife Protection and Hunting Management Law. Monkeys were captured using box traps and then euthanized with a gun by licensed hunters at the request of Fukushima City. The methods for capture and euthanasia were in accordance with the guidelines of the management plan and do not present an ethical concern. This method of euthanasia was also in accordance with guidelines published by the Wildlife Research Center of Kyoto University (2022). The Japanese monkeys inhabiting the study area are not listed as an endangered species on the Japanese Red List (Ministry of the Environment 2020).

### Sample Collection

Japanese monkey carcasses were collected from 2008 to 2022, transported under refrigerated conditions to our laboratory, and subjected to necropsy. The body weight of each monkey was measured in grams. Body length was measured in millimeters as the straight-line distance from the top of the head to the rump at the dorsal surface of the sciatic protuberances while the monkey was in a recumbent position. The uterus and ovaries in females were dissected and fixed in 10% buffered formalin solution after checking for the presence of fetuses.

After the FDNPP accident, 500–1,000 g muscle tissue from the hind limb was collected during the necropsy; this tissue type was used because tissue weighing 500 g or more is needed to measure the radiocesium concentration. The muscle tissue was stored at − 30 °C until it was used for radioactivity measurements.

### Evaluation of the Nutritional Status

The ratio of the mesenteric fat weight to body weight is proportional to the percentage of body fat in Japanese monkeys (Hayama et al. 2017). During the necropsy, nutritional status was evaluated according to the fat index (FI), which is calculated by dividing the mesenteric fat weight (g) by body weight (g) and multiplying by 1,000. Because FI varies seasonally (Hamada et al. 2003), in this study we determined the month with the highest median value during the study period and analyzed individuals captured in that month to examine the relationship between FI and age and the change in this relationship over time.

### Age Determination

The age of each individual was assessed by tooth eruption from 0 to 6 years of age (Iwamoto et al. 1987). Age after 7 years, when all permanent teeth had erupted, was assessed by extracting the upper first incisor and observing the annual rings formed on the root cement layer, following the method of Wada et al. (1978). The year of capture and the age assessed by these methods were used to estimate the year each individual was born. Because the mean date of birth among Japanese monkeys in Fukushima City is estimated to be May 11 (standard deviation ± 29.2 days) (Hayama et al. 2011), in this study, we assumed that all individuals were born in May. The year each individual was born and the date of capture were used to determine the age in months.

### Estimation of Ovulation and Conception Rates

**Fig. 2:**
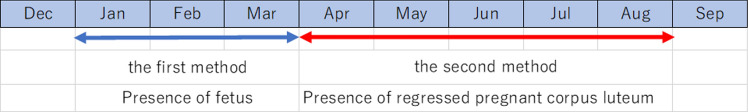
Month of capture and method of determining conception rate in the year of delivery

Japanese monkeys are seasonal breeders, with a mating season from late fall to early winter, an average gestation period of 173 days (Nigi 1976), and a spring delivery season. Japanese monkeys in Fukushima City are able to conceive at the age of 4 years, and deliver their infants as early as spring at the age of 5 years (Hayama et al. 2011). In this study, we estimated the conception rate of individuals who were at least 5 years old in the year of capture during their delivery season (hereafter referred to as “year of delivery”). The following two methods were used to estimate conception rates, depending on the year of delivery (Fig. 2).

The average conception date during the mating season of Japanese monkeys in Fukushima City is estimated to be November 19 (standard deviation ± 29.2 days) (Hayama et al. 2011). Considering the estimated conception and delivery date deviations together, it would be reasonable to determine whether conception occurred between January and March based solely on the presence or absence of fetuses. Therefore, in this study, conception rates in individuals captured between January and March were estimated based on the presence or absence of fetuses, using the first method (Fig. 2).

In Japanese monkeys, the corpus luteum is formed after ovulation, and its remnants are histologically discernible for nearly a year after ovulation (Hayama et al. 1991). Also, as in humans (Mossman and Duke 1973), collagen fibers are prominently developed in the corpus luteum of pregnant monkeys. However, as the estrous corpus luteum regresses, lipofuscin deposits increase in the corpus luteum (Hayama et al. 1992), making it easy to histologically distinguish between the regressed and estrous corpus luteum in the ovaries after the delivery season. In a previous study (Hayama et al. 2011), the earliest conception date for Japanese monkeys in Fukushima City was reported to be September, so using the second method of this study (Fig. 2), conception rates for individuals captured from April to August were estimated by the presence or absence of a regressed corpus luteum in the ovaries. Individuals in which no regressed or estrus corpus luteum was observed were treated as non-ovulated individuals, and their percentage in the sample population (non-ovulation rate) was evaluated.

To histologically examine the regressed or estrous corpus luteum, the ovaries were embedded in paraffin and sectioned into 2 to 4 slices at 4-μm in the usual manner to facilitate observation of the maximally divided surface of the ovary. Serial sections were produced according to the need for observation. The sections were subjected to Masson trichrome staining and observed under an optical microscope.

### Radioactivity Measurements

Muscle radiocesium concentration (Bq/kg) was measured in monkeys captured after the accident, but muscle samples were not collected before the accident. The radioactivity of radiocesium in the muscle samples was analyzed using a germanium semiconductor spectrometer (GC2020-7500SL-2002 CSL; Canberra Industries, Meriden, CT) and a NaI (T1) scintillation detector (AT1320A; Atomtex SPE, Minsk, Belarus). Data were corrected to the background radiation dose in the measurement environment as needed. ^134^Cs was detected using 604.70- and 795.85-keV gamma rays, whereas ^137^Cs was detected using 661.6-keV gamma rays. The radioactivity of radiocesium was adjusted to the value on the day of capture based on its physical half-life. The limit of detection was 10 Bq/kg. The muscle radiocesium concentration was calculated as the combined concentration of ^134^Cs and ^137^Cs per kilogram of fresh muscle.

### Estimation of Cumulative Exposure Dose

To assess the effects of exposure on reproduction, the cumulative exposure dose for each individual was estimated. Urushihara et al. (2018) estimated the radiocesium dose-rate using the ERICA tool (ver. 1.2; Brown et al. 2016), with some modifications, and determined the dose conversion coefficients (DCCs), using the equation provided by the ERICA tool for Japanese monkeys (Supplementary Table S4 in Urushihara et al. 2018). In this study, using the DCCs for Japanese monkeys determined by Urushihara et al. (2018), the daily internal and external exposure doses were calculated for ^134^Cs and ^137^Cs, respectively, after which the cumulative exposure dose from March 14, 2011, when the FDNPP melted down and radioactive materials were released, to the time of capture was estimated for each individual. In this study, we assumed that each female Japanese monkey lived within the capture site throughout the exposure period, given that females spend their entire lives in the troop they are born into and troops rarely move out.

Because DCCs vary according to individual body size, Urushihara et al. (2018) used body length as an index of body size for Japanese monkeys, dividing them into four size categories (with 30, 40, 50, and 60 cm as representative values) and determining the DCCs corresponding to each category. Because Japanese monkeys grow until around 8 years of age (Hamada 1994), it is necessary to determine the relationship between age and body growth for each individual. Therefore, in this study, we devised an equation that relates body length and age in months for females at the time of capture, and determined the range of age in months corresponding to each DCC.

The relationship between female body length (y; cm) and age in months (x) at the time of capture was fitted to the Gompertz curve below, which is an equation for body growth, after which the coefficients K, b, and c, which have the highest correlation coefficient, were determined.

$$y=K {b}^{\left\{{e}^{\left\{-c x\right\}}\right\}}$$

These coefficients were substituted to obtain the monthly ages corresponding to the boundary values of the four categories: 35, 45, and 55 cm in body length.

The cumulative exposure doses were obtained by calculating the monthly exposure doses for each individual during the exposure period and adding them up, as shown below.

The cumulative external exposure dose of captured individuals was calculated based on the soil deposition (Bq/m^2^) of radiocesium (^134^Cs and ^137^Cs) at the capture site as follows. At first, the amount of soil deposition on the first day of each month from the beginning of exposure for each individual to the time of capture was determined from the physical half-life based on monitoring data collected by the Ministry of Education, Culture, Sports, Science and Technology on July 4, 2011. Next, the data for each month were multiplied by the external DCC (µGy/day) / (Bq/m^2^) corresponding to that month’s age to obtain the daily external exposure dose, which was then multiplied by 30 to obtain the external exposure dose for that month. Finally, the sum of the external exposure doses for each month was calculated to obtain the cumulative external exposure dose for the individual.

The cumulative internal exposure dose for each captured individual was calculated based on the aggregated transfer factor (T_ag_; i.e., the geometric mean of the ratio of the radiocesium concentration in the muscle of all individuals captured in a given year to the soil deposition at the capture site of each individual in m^2^/kg) for each year as follows. T_ag_ for each year was assumed to be constant during that year. At first, the soil deposition at the capture site on the first day of each month from the beginning of exposure to capture for each individual was determined in the same way as the calculation of the cumulative external exposure dose. The data were multiplied by the corresponding year’s T_ag_ to obtain the muscle concentration of radiocesium for each month. Next, the muscle radiocesium concentration for each month was multiplied by the internal DCC (µGy/day) / (Bq/kg) corresponding to the age of the month in order to obtain the daily internal exposure dose, which was then multiplied by 30 to obtain the internal exposure dose for that month. Finally, the sum of the internal exposure doses for each month was calculated to obtain the cumulative internal exposure dose for the individual.

### Statistical Analysis

Fisher’s exact test was used for the test of ratio and 95% confidence intervals. The Mann–Whitney *U* test was used to test the difference in medians. For multiple comparisons, pairwise comparisons using the Mann–Whitney *U* test were performed with the Bonferroni correction. The Cochran–Armitage test was used to test for a trend in the ratio, and the Jonckheere–Terpstra test was used to test for a trend in the median. Spearman’s rank correlation was used to test for correlations.

All analyses were performed using Stata Statistical Software Release 16 (StataCorp, College Station, TX). For statistical estimation and inference, two-sided hypotheses and tests were used with a 5% significance level.

## Results

### Conception Rate

**Fig. 3:**
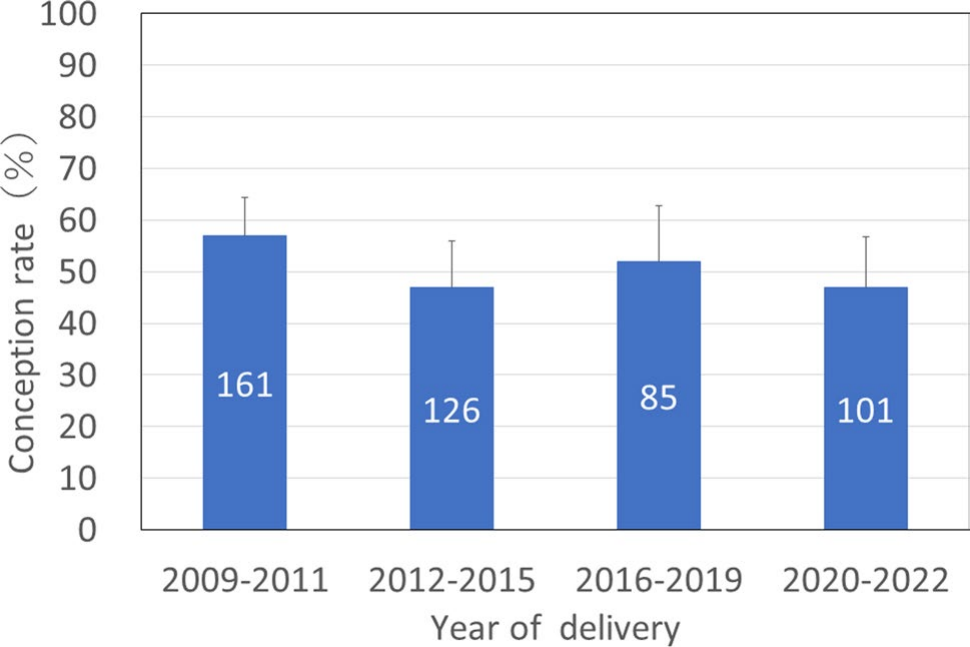
Conception rate (%) by delivery years in Japanese monkeys (n = 473). Error bar indicate 95% confidence interval. Numbers in the bars indicate the sample size

The conception rate was determined by analyzing 473 females that were at least 5 years old in the year they were captured. Multiple comparisons of conception rates by year of delivery were attempted, but statistical tests could not be performed due to the small number of cases in some years. Therefore, we divided the study period into the following four periods in order to examine the differences in conception rates over time. The conception rates for 2009–2011, which were considered to have occurred before the FDNPP accident (“pre-exposure”), were 57% (91 conceived, 70 did not). The conception rates after the FDNPP accident (“post-exposure”) were 47% (59 conceived, 67 did not) for 2012–2015, 52% (44 conceived, 41 did not) for 2016–2019, and 47% (47 conceived, 54 did not) for 2020–2022 (Fig. 3). There were no significant differences in conception rate in these four periods (*P* = 0.264). There was also no significant increasing or decreasing trend over time (*P* = 0.166).

**Fig. 4:**
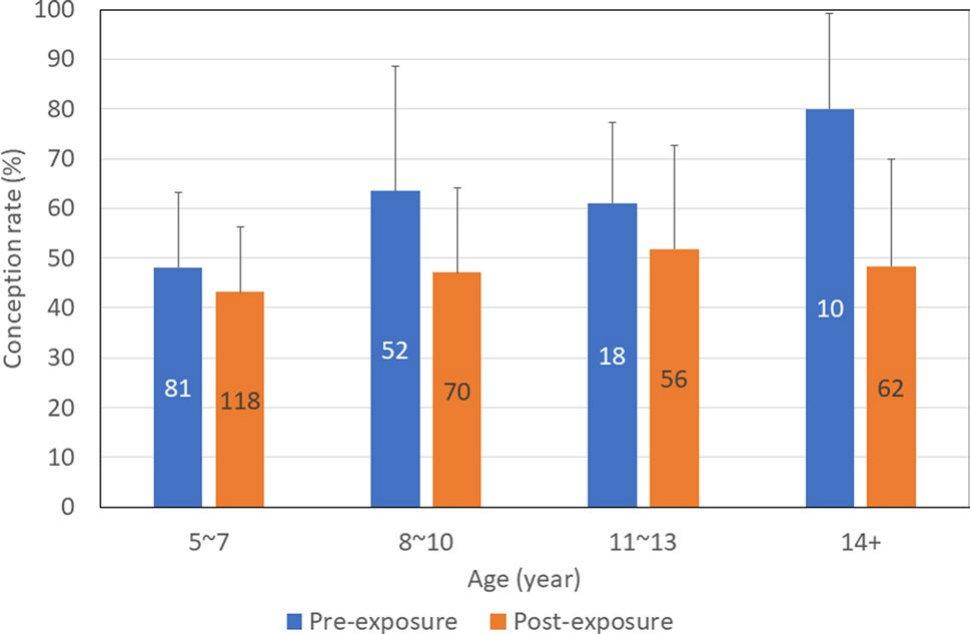
Conception rate (%) by age class with and without radiation exposure in Japanese monkeys (n = 467). Error bar indicate 95% confidence interval. Numbers in the bars indicate the sample size

Conception rates by age were compared between the pre- and post-exposure periods (*n* = 467). Due to the skewed sample size, the analysis was divided into four age classes: 5–7, 8–10, 11–13, and ≥ 14 years. The conception rate tended to increase significantly with age pre-exposure (*P* = 0.004), while neither an increase nor a decrease was significant post-exposure (*P* = 0.235) (Fig. 4).

There was no significant difference in conception rate between the pre- and post-exposure periods for any of the age classes. The P value for the 5–7 year age group was 0.562, while those for the 8–10, 11–13, and ≥ 14 year age groups were all < 0.1; therefore, the conception rates for the ≥ 8 year age groups were compared between the pre- and post-exposure periods and a significant difference was found (*P* = 0.017) (Table 1).

**Table 1:** Median and p-values by age class for conception rate, non-ovulation rate, and January Fat index pre- and post-exposure

**Analysis items by age class**		**Pre-exposure**			**Post-exposure**		**p-value**
Conception rate (%)		n	Value (95% confidence interval)		n	Value (95% confidence interval)	
5–7 years		81	48.1 (36.8–59.5)		118	43.2 (33.7–52.7)	0.562
≥ 8 years		80	65.0 (54.7–75.3)		188	48.9 (41.6–56.3)	**0.017**
Non ovulation rate (%)		n	Value (95% confidence interval)		n	Value (95% confidence interval)	
5–7 years		78	34.6 (24.2–46.2)		58	43.1 (30.2–56.8)	0.373
≥ 8 years		74	17.6 (9.7–28.2)		89	21.3 (13.4–31.3)	0.560
Fat index in January		n	Median (interquartile range)		n	Median (interquartile range)	
5–7 years		18	16.3 (12.5–20.6)		23	15.1 (12.2–19.0)	1.000
≥ 8 years		20	20.0 (16.3–23.5)		34	19.4 (14.5–23.2)	1.000

### Non-Ovulation Rate

Non-ovulation rates by age were compared between the pre- and post-exposure periods. Due to the small sample size, the analysis was divided into two age classes, 5–7 and ≥ 8 years, like the conception rate. The non-ovulation rate was significantly higher in the age class 5–7 years than in the age class ≥ 8 years, regardless of exposure (*P* = 0.026 pre-exposure, *P* = 0.006 post-exposure; Table 1). However, there was no significant difference in the non-ovulation rate between the pre- and post-exposure periods for any age class (*P* = 0.373 for the age class 5–7 years and *P* = 0.560 for the age class ≥ 8 years; Table 1).

### Nutrition Status

**Fig. 5:**
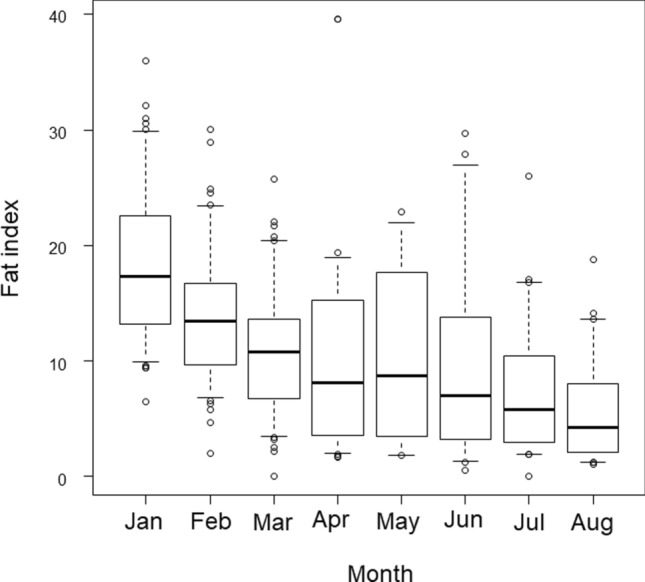
Fat index by month (January to August) in Japanese Monkeys (n = 467). The box plot shows 5th (lower whisker), 25th (bottom edge of the box), 75th (top edge of the box), and 95th (upper whisker) percentiles. The median concentrations are given as the line within the box, and the open circles are outliers

When comparing the seasonal changes in FI during the study period of January through August (*n* = 467), the median value for January was the largest and differed significantly from the medians for all other months (*P* < 0.01, Fig. 5). Therefore, we compared nutritional status over time, using the FI in January of each year, and found no significant differences in the medians of each year (*P* = 0.361). There was also no significant increasing or decreasing trend over time (*P* = 0.601).

The relationship between FI in January and age class was examined in the same way, but due to the small number of cases in some age classes, multiple comparisons of median values were performed for four age classes: 5–7 years (pre-exposure *n* = 18, post-exposure *n* = 23) and ≥ 8 years (pre-exposure *n* = 20, post-exposure *n* = 34). The results showed no significant differences among these four groups (Table 1).

### Cumulative Exposure Dose

**Fig. 6:**
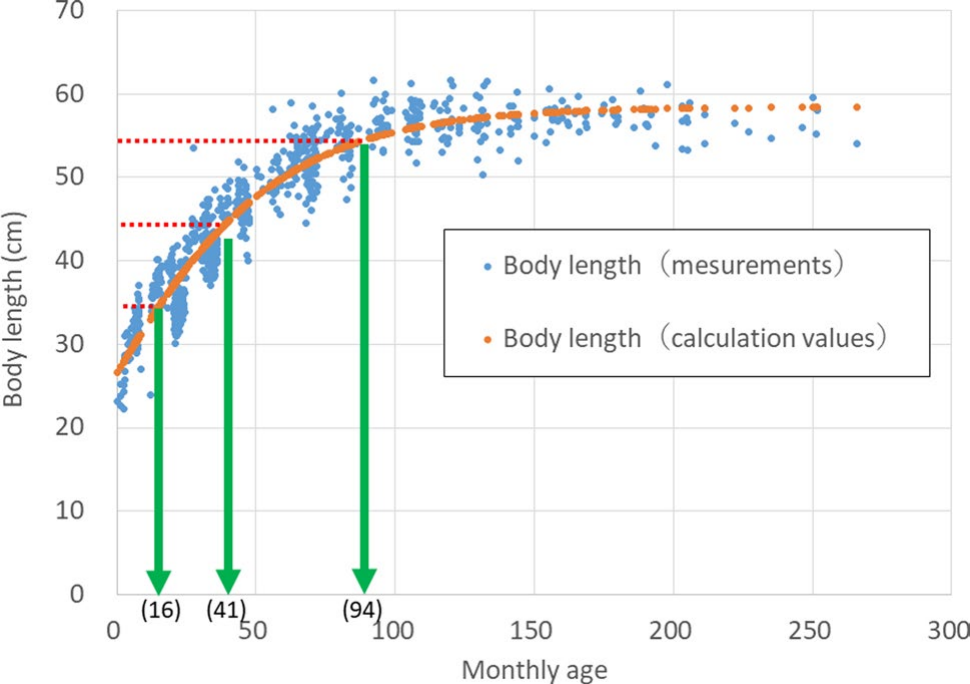
Relationship between body length (mm) and monthly age in female Japanese monkeys (n = 797). Blue dots are measured values and orange dots are calculated values. Based on the relationship between the calculated values, the ages for body lengths of 350, 450, and 550 mm were estimated to be 16, 41, and 94 months, respectively

Data from 797 females of known body length and age in months were fitted to a Gompertz curve to obtain the coefficients of the equation. The obtained coefficients were *K* = 58.45, *b* = 0.453, and *c* = 0.0271. There was a high correlation between the calculated and measured values of body length using the equation (Spearman’s rank correlation coefficient 0.944, *P* = 2.2E-16). Based on this equation, the boundary age in months for the four categories was 16 months for 35 cm body length, 41 months for 45 cm, and 94 months for 55 cm, (Fig. 6, Table 2).

**Table 2:** Dose conversion coefficient (DCC) of each body length (Urushihara et al. 2018, Supplementary Table S4) and range of age in months corresponding to body length (present study) in Japanese monkeys

Range of body length (cm)	Range of age in months (Present study)	Internal DCC (µGy/day) / (Bq/kg)		External DCC (µGy/day) / (Bq/m^2^)	
		^**134**^**Cs**	^**137**^**Cs**	^**134**^**Cs**	^**137**^**Cs**
25–35	0–16	5.41E-03	4.59E-03	1.16E-04	4.25E-05
35–45	17–41	6.46E-03	4.98E-03	1.10E-04	3.98E-05
45–55	42–94	7.47E-03	5.36E-03	1.04E-04	3.79E-05
55–65	95–	8.46E-03	5.72E-03	9.70E-05	3.53E-05

Analyzing 334 females aged ≥ 5 years captured from 2011 to 2022, we determined the T_ag_ for each year corresponding to ^134^Cs and ^137^Cs, which is necessary for estimating cumulative internal exposure doses (Table 3).

**Table 3:** Geometric means of radiocesium by year (2011–2022) in Japanese monkeys

Year	n	Geometric mean of ^134^Cs T_ag_ (m^2^/kg)	Geometric mean of ^137^Cs T_ag_ (m^2^/kg)
2011	32	7.60E-03	8.34E-03
2012	51	3.58E-03	3.72E-03
2013	38	1.85E-03	2.00E-03
2014	20	1.65E-03	1.85E-03
2015	15	1.21E-03	1.52E-03
2016	4	9.79E-04	1.66E-03
2017	8	1.15E-03	1.77E-03
2018	26	1.15E-03	1.16E-03
2019	41	3.33E-04	8.75E-04
2020	39	3.39E-04	1.31E-03
2021	30	1.21E-04	5.58E-04
2022	30	1.51E-04	6.85E-04

Based on these data, we calculated the cumulative internal exposure dose and the cumulative external exposure dose for each individual, analyzing 302 females that were ≥ 5 years of age and had known capture dates in the year of delivery since 2012. The median cumulative internal exposure dose was 4.2 m Gy (minimum 0.2, maximum 12.4) and the median cumulative external exposure dose was 12.5 m Gy (minimum 1.0, maximum 42.4). The cumulative internal and external exposure doses for each individual were significantly correlated (Spearman’s rank correlation coefficient 0.81, *P* = 1.54E-71). Therefore, the cumulative internal and external exposure doses for each individual were summed to obtain the total cumulative exposure dose.

**Fig. 7:**
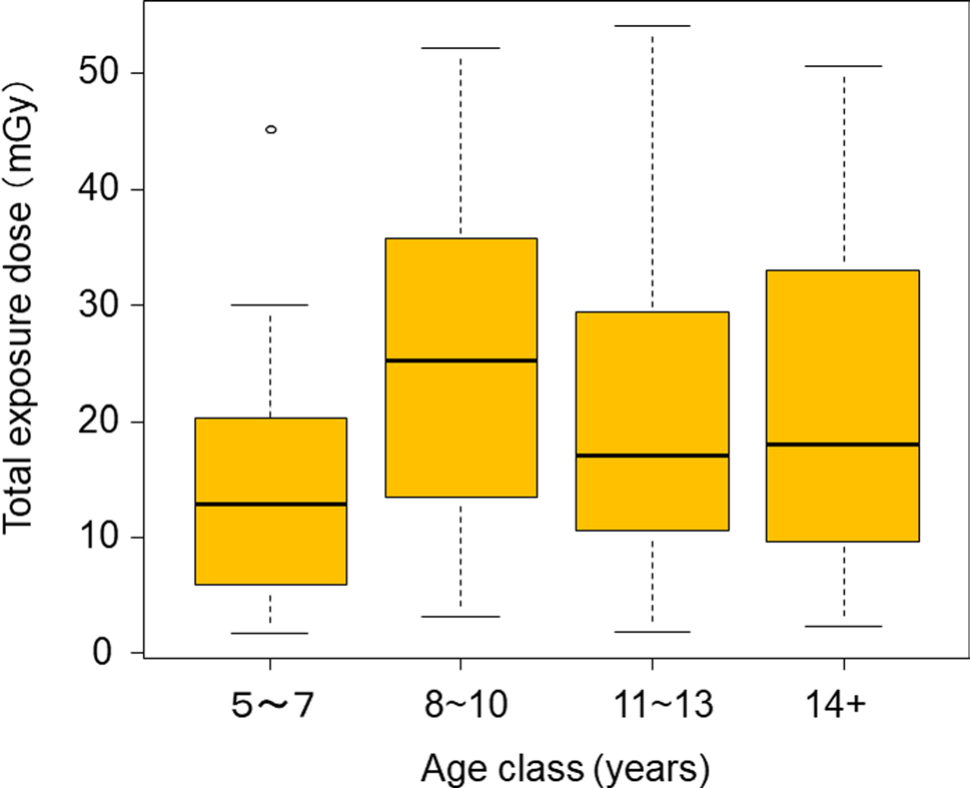
Total exposure dose (mGy) by age class in Japanese monkeys (n = 302). The box plot shows 5th (lower whisker), 25th (bottom edge of the box), 75th (top edge of the box), and 95th (upper whisker) percentiles. The median doses are given as the line within the box and the open circles are outliers

Comparing the median total cumulative exposure dose by age class, the median dose was significantly higher for the age classes 8–10 years, 11–13 years, and ≥ 14 years at 25.2 mGy, 17.0 mGy, and 18.1 mGy, respectively, compared with 12.8 mGy for the age class 5–7 years, with P values of 4.5E-7, 9.6E-3, and 0.021, respectively (Fig. 7).

## Discussion

We examined changes in the conception rates of wild Japanese monkeys in Fukushima City from 2009 to 2022, including those exposed to radiation due to the accident at the Fukushima Daiichi Nuclear Power Plant (FDNPP). There were no significant differences in conception rates over time, and there appears to have been no effect of exposure due to the 2011 FDNPP accident in this population.

The conception rate by age tended to increase significantly with age during the pre-exposure period. Previous studies on reproduction in Japanese monkeys have reported an increase in the conception rate with age based on direct observation in specific troops (Itoigawa et al. 1992; Koyama et al. 1992; Takahata et al. 1998). The present study confirmed a similar phenomenon in this wild population. However, during the post-exposure period, there was no significant difference in conception rates by age. In addition, there was no significant difference in conception rate between the pre- and post-exposure periods in the age class 5–7 years, but there was a significant decrease in the conception rate post-exposure compared with pre-exposure in the age class ≥ 8 years. This suggests that the conception rate in the age class ≥ 8 years declined after exposure for some reason.

In provisioned troops of Japanese monkeys, the birth rate increases with the amount of food provided (Sugiyama and Ohsawa 1982). This is because increased body fat ratio is associated with reproductive success (Garcia et al. 2011). Even in wild troops, it has been reported that birth rates vary depending on the abundance of acorns, and a relationship with body fat ratio has been postulated (Noma et al. 1998). However, no studies have demonstrated a relationship between body fat ratio and reproductive success in wild troops. This is probably due in part to the difficulty of obtaining data from wild individuals as well as the difficulty of clarifying the relationship between body fat ratio, which varies widely and seasonally, and the timing of conception.

FI was measured during the study period, January through August, and it was found that FI was highest in January, which was considered an appropriate time to examine the relationship between conception rate and nutritional status for the year, given that January is when most of the individuals had conceived. Although this limited time period reduced the overall sample size, it was deemed feasible to compare conception rates over time and by age class. No significant differences were found in FI between the pre- and post-exposure periods or in FI by age class. Thus, a relationship between conception rates and nutritional status could not be clearly established for the time period covered in this study, and there was no evidence that changes in nutritional status had an effect on the decline in conception rates for the age class ≥ 8 years in the post-exposure period.

This study focused on cumulative exposure dose as a factor affecting conception rates and attempted to estimate it for each individual animal. Studies on the effects of long-term low-dose exposure on reproduction in humans and laboratory animals are scarce, and it is impossible to determine the range of behavior related to external exposure dose as well as the eating habits related to internal exposure dose over the lifetime of wild animals, which are data necessary for estimating cumulative exposure dose. Thus, several assumptions must be made. The three main assumptions in this study are as follows: (1) all individuals were born in May, (2) they lived their entire lives within the capture site, and (3) there were no individual differences in body growth.

Regarding (1), because the deviation in the conception date in this area is about 2 months (Hayama et al. 2011), there should be no considerable impact on the calculation of cumulative exposure doses over several years. As for (2), given that female Japanese monkeys live their entire lives in their natal troop and there is no evidence to suggest that the troop’s home range changed substantially during the study period, there should be no considerable impact on the calculation of cumulative exposure doses over several years. In addition, Urushihara et al. (2018) used the same method to calculate external exposure dose, comparing the soil accumulation concentration at the capture site with the average soil accumulation concentration within a 30-km radius, which is the home-range area for Japanese monkey troops, and adopted the soil accumulation concentration at the capture site because a significantly high correlation was obtained. In the present study, the external exposure dose was also calculated according to this finding. As for (3), the relationship equation between body length and age in months was obtained using only females captured in the study area, and given that a significantly high correlation was obtained between the measured and calculated values, it is considered that there was no considerable influence on the calculation of cumulative exposure doses.

In this study, the total cumulative exposure was compared by age class. The total cumulative exposure dose by age class was significantly higher for the age class ≥ 8 years than for the age class 5–7 years. The fact that there was no significant difference in conception rates between the pre- and post-exposure periods for the age class 5–7 years, while there was a significant decrease in conception rates post-exposure compared with pre-exposure for the age class ≥ 8 years, may be related to the difference in total cumulative exposure dose.

There are many studies on the effects of radiation exposure on reproduction. Skrzypek et al. (2019) reviewed studies on the effects of ionizing radiation on reproduction in women and noted a dependency on dose fractionation and age. In other words, older women are considered to be more radiosensitive, and this is related to the pool of ovarian follicles available at different ages. Accordingly, it was considered that the lower conception rate of older individuals was due to an increase in the number of non-ovulating individuals resulting from the death of oocytes. However, in the present study, a comparison of the ratio of non-ovulatory individuals in the most recent ovulation period by age class showed that the ratio was significantly lower in the age class ≥ 8 years than in the age class 5–7 years, in both the pre- and post-exposure periods. Furthermore, a comparison of the anovulation rate between the pre- and post-exposure periods for each age class showed no significant difference in any of the age classes. Therefore, the effects of increasing age and exposure on the anovulation rate were considered to be unrelated.

If the decrease in conception rate is not due to an increase in the non-ovulation rate, some abnormality after ovulation may be the cause. Hansmann et al. (1982) irradiated pre-ovulatory mouse oocytes with various doses of X-rays (0.05–0.80 Gy) and recorded the rate of non-separation during the first meiotic division and chromosomal structural abnormalities in ovulated oocytes in the mid-II phase. The results showed that although there was no statistically significant increase in disjunctions after low-dose irradiation, chromosomal structural aberrations occurred even at radiation doses as low as 0.05 Gy. It is possible that these chromosomal structural abnormalities in the oocytes may have affected the conception rate after ovulation.

Wo et al. (2009) reviewed studies on the reproductive effects of radiation therapy in women with cancer and found that craniospinal irradiation causes significant hormonal changes in women that affect their subsequent ability to conceive, and that women treated with abdominal pelvic radiation therapy have higher rates of miscarriage, premature birth, low birth weight, and uterine dysfunction leading to placental abnormalities. Chiarelli et al. (2000) also analyzed the risk of adverse pregnancy and neonatal outcomes in 340 female cancer survivors after abdominopelvic irradiation and reported that infant weight and perinatal infant mortality as well as the likelihood of low birth weight were significantly associated with radiation dose, and they concluded that these findings were the result of radiation-induced uterine damage. In Japanese monkeys in Fukushima City, another study found that fetal weight gain was delayed post-exposure compared with pre-exposure, and the delay was correlated with the mother’s relative radiation dose (Hayama et al. 2023). Although the results cannot easily be compared with those of Chiarelli et al. (2000) because of the different exposure doses and exposure situations, the lower conception rate in the present study may be attributable to some uterine effects. Because hormone measurements and histopathological observations of the uterus were not performed in this study, a future study should examine the relationship between these factors and conception rates.

## Conclusions

The conception rate of Japanese monkeys in Fukushima City was estimated by analyzing captured individuals, and its trends over time, relationship with age, and effects of radiation exposure were examined. This is the first report examining the reproductive effects of a nuclear disaster on large mammals. The conception rate tended to increase significantly with age in the pre-exposure period from 2009 to 2011, but this trend was not observed in the post-exposure period from 2012, and the conception rate significantly decreased in the age class ≥ 8 years in the post-exposure period compared with the pre-exposure period. FI in January was used as an indicator of nutritional status, and no significant differences were found when comparing age classes between the pre- and post-exposure periods. The non-ovulation rate was also compared between the pre- and post-exposure periods for each age class, and no significant differences were found. However, the total cumulative exposure dose was significantly higher for the age class ≥ 8 years than for the age class 5–7 years.

These results suggest that the cumulative exposure dose may be one of the reasons for the lower conception rate for the age class ≥ 8 years in the post-exposure period.

## Data Availability

Data will be made available on request.
